# Suicide versus homicide firearm injury patterns on trauma systems in a study of the National Trauma Data Bank (NTDB)

**DOI:** 10.1038/s41598-022-17280-2

**Published:** 2022-09-19

**Authors:** Christopher W. Foote, Xuan-Lan Doan, Cheryl Vanier, Bianca Cruz, Babak Sarani, Carlos H. Palacio

**Affiliations:** 1 McAllen Medical Center, South Texas Health System, McAllen, TX USA; 2 General Surgery Department, Graduate Medical Education, Valley Health System, Las Vegas, NV USA; 3 School of Medicine, Touro University, Henderson, NV USA; 4 Trauma Department, George Washington University, Washington, DC USA; 5 Pharmacy Department, South Texas Health System, McAllen, TX USA; 6 Trauma Department, South Texas Health System, McAllen, TX USA

**Keywords:** Health policy, Public health, Epidemiology

## Abstract

Firearm related mortality in the USA surpassed all other developed countries. This study hypothesizes that injury patterns, weapon type, and mortality differ between suicide groups as opposed to homicide. The American College of Surgeons National Trauma Database was queried from January 2017 to December 2019. All firearm related injuries were included, and weapon type was abstracted. Differences between homicide and suicide groups by sex, age, race, and injury severity were compared using a Mann–Whitney test for numerical data and Fisher’s exact test for categorical data. The association between weapon type and mortality relative to suicide as opposed to homicide was assessed in Fisher’s exact tests. Significance was defined as *p* < 0.05. There were 100,031 homicide and 11,714 suicide subjects that met inclusion criteria. Homicides were mostly assault victims (97.6%), male (88%), African–American (62%), had less severe injury (mean (ISS) 12.07) and a median age of 20 years old (IQR: 14, 30, *p* < 0.01). Suicides were mostly male (83%), white (79%), had more severe injury (mean ISS 20.73), and a median age of 36 years old (IQR: 19, 54, *p* < 0.01). Suicide group had higher odds of head/neck (OR = 13.6) or face (OR = 5.7) injuries, with lower odds of injury to chest (OR = 0.55), abdominal or pelvic contents (OR = 0.25), extremities or pelvic girdle (OR = 0.15), or superficial soft tissue (OR = 0.32). Mortality rate was higher for suicide group (44.8%; 95% confidence interval (CI) 43.9%, 45.7%) compared to the homicide group (11.5%; 95% CI 11.3%, 11.7%). Suicide had higher mortality, more severe injuries, and more head/neck/facial injuries than homicide. Majority of suicides were with handguns.

## Introduction

Firearm related injury is a complex public health issue worldwide. While firearm death rates declined from 2003 to 2015 among most high-income countries, the United States (US) death rates increased from 10.2 to 11.2 per 100,000 people^1^. In 2015, the overall US firearm death rate was 11.4 times higher than 28 other high income countries combined, thereby giving the US the highest rates of firearm homicide, firearm suicide, and unintentional firearm related deaths per 100,00 people^1^. Firearm homicide and suicide are among the top ten causes of injury deaths in the US. In 2018 there were 38,390 firearm deaths, with 24,432 firearms suicide (50.5% of all suicides) and 13,958 firearm homicides (74.1% of all homicides) which, combined, surpassed unintentional falls and motor vehicle collisions. In 2019, firearm homicide and suicide was the leading cause of violence-related injury death among persons 15–34 years of age^2^. Suicide and homicide accounted for 8.1% and 5.1% years of potential life lost, respectively, before the age of 65 in 2019^2^.

Firearm injuries may be preventable, however, the issues are multi-faceted. Violence by firearm occurs in a variety of ways and can have a lasting effect on the community suffering that violence^3–8^. Survivors of firearm injuries can be witnesses, responders, family, and of course the injured. As clinicians, we have a particular opportunity to affect the patient and communities we serve in both primary and secondary prevention^9,10^. Access to firearms and inadvertent discharge have been readily discussed in the literature as well as the strategy of primary prevention by way of “means safety”^6–8,10–34^. This has been reported as having an indirect effect^15,16^ and combined with media reporting practices as primary prevention strategies, are outside the realm of healthcare clinicians. Primary prevention strategies that can be affected by clinicians such as community awareness programs, crisis intervention, and access to appropriate healthcare services have shown direct benefits, per the World Health Organization and several multicenter studies^9^.

Secondary prevention practices involving direct care to the injured by “gatekeepers”^9^ to treat the acute traumatic injury as well as provide “physical, psychological, and emotional safety” and “a sense of control and empowerment”^8^. Intentional firearm injury that does not immediately lead to death, whether they die as an inpatient or survive to discharge, comes with significant morbidity, recovery, and impact on social and interpersonal needs of the patient. Failed suicide attempt, non-suicidal self-harm, assault, and injury as a result of law enforcement/military/war actions impart a burden on healthcare and community resources. In an attempt to advance injury prevention practices we seek to gather, analyze, and disseminate data on the nature and extent of the problem. This study seeks to compare the differences in population, substance use, injury patterns, weapon type, and in-hospital mortality between suicide and homicide groups that survive to reach trauma centers for care using a large nationally reported trauma database. As direct patient healthcare providers we can identify those patients that could benefit from additional resources with multidisciplinary medical care and community resources in addition to treating the primary injury.

## Methods

A retrospective study from January 2017 to December 2019 was performed using the American College of Surgeons (ACS) National Trauma Data Bank (NDTB)^35^. All methods were performed in accordance with the relevant guidelines and regulations. This study does not require any approval by an Institutional Review Board as the NTDB contains only publically available, de-identified data compiled from millions of electronic records out of hundreds of trauma centers across the US. The NTDB collects data from patients who present to all level I and level II trauma centers. Level III and IV trauma centers can participate in the data bank voluntarily. All data are de-identified and aggregated, and searchable through the American College of Surgeons. With more than 7.5 million electronic records, the NTDB is the world’s largest trauma data repository^36^ and the most robust source of clinical information for firearms injury^37^.

All firearm related injuries and weapon type were abstracted using ICD-10 codes (W32-W34.XXX Firearm injury: Unintentional (unintentional shooting, fatal or non-fatal), X72-74.XXX Firearm injury: Self-harm (gun suicide, attempted or completed), X93-X95.XXX Firearm injury: Assault (gun homicide, attempted or completed), Y22-24.XXX Firearm injury: Undetermined intent (unknown cause, fatal or non-fatal), Y35.XXX Firearm injury: Justifiable shooting (legal intervention, fatal or non-fatal), Y36.4XX Firearm injury: War operations (war shooting, fatal or non-fatal). Division of groups were made for comparison simplicity with ‘Homicide’ including firearm injuries inflicted onto the patient, and ‘Suicide’ including firearm injuries that were purposely self-inflicted.

The demographic information and injury severity of homicide and suicide groups were compared using a Mann–Whitney test for numerical data and Fisher’s exact test for categorical data. Logistic regression was used to determine the ways that injury location, injury type/severity, and detected drugs were associated with suicide as opposed to homicide. The association between weapon type and mortality relative to suicide as opposed to homicide was assessed in Fisher’s exact tests. Significance was defined as *p* < 0.05.

### Consent for publication

All authors have read and approved final version of this work. No waiver of consent was necessary from patients as was de-identified data review.

## Results

During the study period, 100,031 homicides and 11,714 suicides were identified. The homicide group was composed of individuals who were categorized as assault (n = 97,639), legal intervention (n = 2,303), military operations (n = 9), terrorism (n = 76), or war operations (n = 4). The suicide group included the Intentional Self-Harm categories as described above (n = 11,714). Accidental Discharge (n = 18,920), Accidental Malfunction (n = 397), and Undetermined Intent (2748) categories were not included since we were evaluating causes for intentional firearm injuries.

Demographics between the two groups differed in several ways. Median age for suicide subjects (36, Interquartile range (IQR): 19, 54) was 16 years older than for homicide subjects (20, IQR: 14, 30) (Table 1). African–American subjects made up 62% of the homicide group and only 10% of the suicide group. Asian, American Indian, and White subjects had higher percentages in the suicide group than the homicide group (Table 1). Males comprised the vast majority of both the homicide and suicide groups, 88% and 83%, respectively.

**Table 1:** Demographics about subjects included in the study.

	Homicide		Suicide		*p* value
**Number of subjects**	100,031		11,714		
Age (median, IQR)	20 (14, 30)		36 (19, 54)		< 0.001
Maximum AIS severity	3 (2, 3)		5 (3, 5)		< 0.001
**Sex (%female)**	12.0%	12,233	17.0%	2044	< 0.001
Asian	0.7%	675	1.0%	122	< 0.001
Pacific Islander	0.2%	234	0.2%	24	0.611
Raceother	9.1%	9085	5.3%	619	< 0.001
American Indian	0.7%	657	1.1%	125	< 0.001
African American	62.3%	62,331	10.1%	1178	< 0.001
White	24.9%	24,877	78.9%	9246	< 0.001
Race unavailable	1.0%	970	1.2%	144	0.009
Race unknown	1.7%	1682	2.5%	297	< 0.001

The percent and number of subjects in each category are provided, and for age and maximum severity, the median and interquartile range (IQR) are reported for each group.

A comparison of Abbreviated Injury Scale (AIS) scores between both groups demonstrated more severe injuries within the suicide group (5, IQR: 3, 5) than the homicide group (3, IQR: 2, 3) (Table 1). Superficial soft tissue injuries were the most common pattern of injury between both groups (suicide 40%, homicide 67%), however, some significant differences emerged between the two groups (Table 2). Higher rates of injuries to the extremities or pelvic girdle were observed among homicide subjects (34% and 25% respectively, vs. 7% and 8% in suicides), while higher rates of injuries to the head or neck were observed among suicide subjects (67% vs. 13% in homicides). Considering head or neck injuries occurred most frequently among suicide subjects, a greater frequency (despite greater incidence in homicide) of a Glasgow Coma Scale (GCS) less than 13 (19.9 vs. 4.2%), respiratory rate less than 10 or more than 29 (breaths per minute) (8.2% vs. 2.2%), having low systolic blood pressure (6.5% vs. 3.0%), and skull fracture (4.8% vs. 0.3%) would be expected in this group (Table 3). The homicide subjects had higher penetrating injuries indicative of multiple gunshot wounds, greater incidence (despite low overall frequencies) of long bone injury (86 vs. 2), crush injury (173 vs. 9), amputation (29 vs. 1), and paralysis (223 vs. 7).

**Table 2:** Location of injuries for homicide and suicide groups.

	Odds ratio	95% CI	Suicide		Homicide	
Head or neck	13.58	(12.79, 14.42)	7904	67%	13,253	13%
Face	5.72	(5.38, 6.07)	3904	33%	8044	8%
Chest	0.55	(0.52, 0.58)	1800	15%	24,852	25%
Abdominal or pelvic contents	0.25	(0.23, 0.27)	930	8%	25,420	25%
Extremities or pelvic girdle	0.15	(0.14, 0.16)	836	7%	33,711	34%
Superficial soft tissue	0.32	(0.31, 0.34)	4660	40%	67,091	67%

The odds ratio (OR) and its 95% confidence interval (CI) are shown, along with the number and percent of homicides and suicides who had injuries in each location.

**Table 3:** Details and severity of injuries for homicide and suicide groups.

	Odds ratio	95% CI	Suicide		Homicide	
GCS ≤ 13	5.59	(5.16, 6.05)	2327	19.9%	4249	4.2%
SBP < 30	2.24	(2.01, 2.50)	766	6.5%	3026	3.0%
10RR29	3.98	(3.57, 4.44)	961	8.2%	2196	2.2%
Pen	1.38	(1.25, 1.53)	4363	37.2%	30,044	30.0%
Chest	2.16	(1.34, 3.47)	25	0.2%	99	0.1%
Longbone	0.20	(0.05, 0.83)	2	0.0%	86	0.1%
Crushed	0.44	(0.22, 0.89)	9	0.1%	173	0.2%
Amputation	0.29	(0.04, 2.28)	1	0.0%	29	0.0%
Pelvic	0.89	(0.34, 2.35)	5	0.0%	48	0.0%
Skullfracture	18.60	(15.78, 21.82)	568	4.8%	274	0.3%
Paralysis	0.27	(0.12, 0.58)	7	0.1%	223	0.2%

The odds ratio (OR) and its 95% confidence interval (CI) are shown, along with the number and percent of subjects within homicide and suicide groups for each. GCS (Glasgow Coma Scale), SBP (Systolic Blood Pressure), 10RR29 (abnormal respiratory rate less than 10 or greater than 29), PEN (Penetrating injury).

Homicide and suicide group subjects also differed by substances found in their system (Table 4). Suicide subjects most commonly had no substances in their system (OR 2.27, 95% CI 2.16, 2.39). When illicit/recreational drugs were involved, suicide subjects were more likely to have barbiturates (OR 2.82, 95% CI 2.08, 3.81), benzodiazepines (OR 1.61, 95% CI 1.47, 1.77), tricyclic antidepressants (OR 2.47, 95% CI 1.53, 3.98), ecstasy (MDMA) (OR 1.6, 95% CI 1.04, 2.46), or ‘other’ drugs (OR 1.38, 95% CI 1.05, 1.81) in their system. Illicit/recreational drugs such as amphetamines (OR 0.83, 95% CI 0.75, 0.92), cocaine (OR 0.67, 95% CI 0.60, 0.75), opioids (OR 0.71, 95% CI 0.62, 0.81), phencyclidine (PCP) (OR 0.49, 95% CI 0.33, 0.74), and cannabinoids (OR 0.52, 95% CI 0.49, 0.56) were less likely observed in the suicide compared to the homicide group with the most common being cannabinoids.

**Table 4:** Drugs observed in homicide and suicide groups.

	Odds ratio	95% CI	Suicide		Homicide	
Amphetamine	0.83	(0.75, 0.92)	591	5.0%	5999	6.0%
Barbiturate	2.82	(2.08, 3.81)	115	1.0%	351	0.4%
Benzodiazepines	1.61	(1.47, 1.77)	796	6.8%	4326	4.3%
Cocaine	0.67	(0.60, 0.75)	464	4.0%	5798	5.8%
Methamphetamine	0.97	(0.80, 1.17)	179	1.5%	1576	1.6%
Ecstasy	1.6	(1.04, 2.46)	56	0.5%	300	0.3%
Methadone	1.42	(0.88, 2.29)	31	0.3%	186	0.2%
Opioid	0.71	(0.62, 0.81)	320	2.7%	3810	3.8%
Oxycodone	0.9	(0.69, 1.19)	70	0.6%	661	0.7%
Phencyclidine	0.49	(0.33, 0.74)	32	0.3%	554	0.6%
TCA	2.47	(1.53, 3.98)	28	0.2%	97	0.1%
Cannabinoid	0.52	(0.49, 0.56)	1348	11.5%	20,002	20.0%
Other	1.38	(1.05, 1.81)	68	0.6%	421	0.4%
None	2.27	(2.16, 2.39)	2711	23.1%	11,713	11.7%

The odds ratio (OR) and its 95% confidence interval (CI) are shown, along with the number and percent of homicides and suicides who had a drug in their system. *TCA* tricyclic antidepressants.

In-hospital mortality rate was significantly higher (OR = 6.2, *p* < 0.001) within the suicide group (44.8%, 95% CI 43.9%, 45.7%) compared to the homicide group (11.5%, 95% CI 11.3%, 11.7%). Total in-hospital mortalities were still greater with homicides 11,544 versus 5249 indicating that despite greater mortality odds for suicide, burden still high with overall firearm homicide injury victims. Most homicide and suicide incidents involved a handgun or an unspecified firearm (Table 5). In the suicide group, overall in-hospital mortality rates were high, between 44 and 50% for firearms and handguns versus air guns and shotguns at 4–29% (Table 5). This is a stark contrast to the homicide group with an overall in-hospital mortality rate of 11.5% regardless of weapon type.

**Table 5:** Groups differ in the involvement of weapon types and resulting mortality by group.

	Homicide			Suicide			*p* value
	Total Incidents	Mortalities	% mortality (%)	Total Incidents	Mortalities	% mortality (%)	
Handgun	36,555	4075	11.1	7735	3602	46.6	< 0.001
Other firearm	3,353	399	11.9	454	200	44.1	< 0.001
Unspecified firearm	56,036	6795	12.1	2342	1165	49.7	< 0.001
Shotgun	1719	123	7.2	558	116	20.8	< 0.001
Hunting rifle	356	36	10.1	351	103	29.3	< 0.001
Airgun	685	4	0.6	99	4	4.0	0.013
Weapons_other	1327	112	8.4	175	59	33.7	< 0.001
	100,031	11,544	11.5	11,714	5249	44.8	

The number of incidents resulting in mortality and the percent within the group and weapon type, are reported. *p* Values are from Fisher's exact tests to test differences in mortality between groups for each weapon type.

## Discussion

This retrospective study adds to the body of literature concerning gunshot wounds by analyzing data from the NTDB from all trauma centers from within the country representing the highest rates of firearm deaths. In observing the data from patients who arrive to trauma centers alive, we might glean information that may direct us in helping those who can benefit from clinician intervention. Similar to previous publications, we found the suicide group comprised of mostly older white males. A mortality study performed by Branas et al. found most of the suicides were male, older (> 35 years), white, and at least half were unmarried^38^. Their homicide group was also comprised of younger, African–American males^38^. A study on gun violence by Manley et al. also discovered the majority of their patients injured with a documented firearm were African–American males, with a median age of 28, and aggravated assault as the most common circumstance^39^. A retrospective study on interpersonal violence at a Level I trauma center by Livingston et al. also found a significant and disproportionate representation by young African American males^40^. Suicides rates have been associated with white males and rural settings, with homicides rates having been associated with African–American males and urban settings, likely due to the urban–rural divide^34,41^. Urban environments have higher levels of income inequality, female-headed households, crowding^41^, crime, and higher nighttime activity with people coming and going^38^. Rural environments have greater daytime activity, lower traffic of those not living in that neighborhood^38^.

There are multiple modifiable risk factors for firearm violence stemming from motivation and opportunity^41^. Violent firearm crime occurs when three elements converge: suitable targets, motivated offenders, and absence of capable guardians^38^. Larger urban environments provide opportunity, whereas the socioeconomic, cultural, and transitional circumstances provide motivation. Suicide risk is derived from the desire to die and the capability to act^42,43^. Desire to die may arise from depression^42^, antisocial tendencies^44^, social isolation/perceived burdensomeness/thwarted belongingness^42,44^, remorse^6,7^, or a number of other causes leading to dysregulation of emotion^42,44,45^. Suicidal capability may be attained by access to means, inhibition through substance use^46,47^, repeated non-suicidal injury^42^ or self-harm reinforcing pain tolerance^45^, or acute increasing level of pain suffered^42,43^. Firearm-related injuries are a significant public health crisis in the US, contributing to disability, loss of productivity, and tragically, death^3–7,22,48,49^.

This retrospective analysis of the severity of injury and in-hospital mortality found the suicide group experienced more severe injuries in addition to increased mortality compared to the homicide group. Due to the driving factors of desire to die and capability, suicidal actions are deliberately taken for effectiveness. Suicide attempts therefore, as expected, have a high case fatality rate of nearly 90%^50^. An epidemiological trend study on firearm mortality in the US by Goldstick et al. evaluated data from 2006 to 2014, and noted that suicides were consistently the majority of firearm related deaths ranging from 54.6 to 63.7%, respectively^34^. In this study, head and neck injuries were more commonly associated with suicide. Self-inflicted gunshot wounds are more likely to involve the head and neck regions and often result in death^51^. Firearm related injuries to the head are a leading cause of TBI related morbidity and mortality, with one third of patients arriving to the hospital alive, ultimately succumbing to their injury^48^. The remaining two thirds of these will then go on to have TBI with potentially prolonged rehabilitation, motor deficits, cognitive disability, or other neurologic sequelae that will worsen the quality of their daily lives after attempting to end it. Mortality therefore underestimates the full impact and total health effects. Chronic pain, chronic disability, disfiguration, change in personality or mood due to organic or structural changes, risk of infection and need for persistent long-term medical care become a significant part of the both the survivor and loved ones’ interact with their communities^49^.

In this study, extremity injuries were more commonly observed in the homicide group. This group will also likely need additional rehabilitation, possible prosthesis, and will impact their functionality. Fowler et al., described similar results for patients who arrived to the hospital due to unintentional circumstances^48^. Handguns account for most of the firearm related injuries in this retrospective study. Handguns rather than shotgun or semi-automatic have a better survival rate due to continually improving trauma care and single rather than multiple penetrating wounds but still incur a significant burden^22,49,52^. A study evaluating homicidal gunshot wounds at a single Level 1 trauma center in Kansas found handguns to be the most common weapon of choice in their patients^53^. Another study in a rural Midwest level 1 trauma center performed by Guetschow et al. also observed handguns to be the most common weapon type for unintentional firearm related injuries^54^. This trend of handgun use over other types of firearms could mean overall improvement in trauma care as complex injury patterns are significantly more lethal^22,52^ regardless of improved trauma care.

A recent study from the National Violent Death Reporting System (NVDRS) found that 54% of suicide victims did not have a known mental illness^37^. This retrospective study found that detectable antidepressants had much higher odds (OR 2.47, 95% CI 1.53, 3.98) in the suicide group than homicide. When substances were associated with the suicide group, the drugs of choice seem to be related to possibly clinically prescribed or self-medicating for distress via the sedative/analgesic/anxiolytic/antidepressant classes of drugs. This could indicate that these patients may have been already receiving some therapy or were in need of formal therapy. Access to therapists and mental health resources may have been lacking due to full patient loads, low therapist coverage, or a number of other potentially modifiable factors from the healthcare community. Korosec, et al. describes using a density of psychiatrist availability and antidepressant/anxiolytic ratios as a correlation to reduction in suicide rates^55^. Access to highly lethal means, in the setting of acute life stressors and substance use, even without prior suicidal ideation, may explain the increase risk of impulsivity and suicide by firearm^37^.

Conversely, risk factors for homicide include involvement in gang/illegal activity^41^ and illegal firearm possession along with socioeconomic risk factors such as income and social inequality^37^. Living in a community with or having witnessed firearm violence is a risk factor for adverse mental health, behavioral problems, cognitive outcomes, and worsening socioeconomic factors further propagating gang violence^3,4^. Physiological stress and biochemical change result from knowledge of nearby violence with firearm violence significantly impacting the degree of perceived violence and fear^5^. Our study found the homicide group was more likely to have used non-clinical illicit/recreational drugs associated with criminal use. Initiating substance use counseling on index admission rather than having planned follow-up as outpatient after discharge may reduce subsequent recurrence. Social services, psychiatric services, outreach, directed follow-up, and community involvement practices may benefit not only the patient but the unseen victims as witnesses, family, or community members affected by the violence^3–5,8–10,55,56^. Proximity to firearm violence is associated with developing depression, anxiety, and general distrust of social control especially in mothers whose mental health effects spread to her children and home life^5^. Psychiatric services with possible initiation of appropriate antidepressants in suicidal patients in concordance with substance use counseling^8–10,55,56^ may benefit those who were missed, self-medicating, undiagnosed, or needing appropriate care while recovering from their injuries might also improve recovery and long-term outcomes.

Access to firearms has been shown, and well described in the literature, to be associated with increased risk factor for firearm suicide related injuries^25,26,30,31,57,58^. In fact, after controlling for confounding factors such as unemployment, mental illness, poverty, illicit drug and alcohol dependence, firearm availability increases the risk of suicide^26,58,59^. A meta-analysis on accessibility of firearms and risk for suicide and homicide reported a significant increase in the odds of suicide among patients with access to firearms compared to those who did not^26^. Background checks are an evidence-based practice that reduce firearm violence and mortality^15,24^. Healthcare providers can have a positive impact in preventing firearm related injury by intentionally engaging in discussion with patients about firearm ownership and safety^26,57,58^.

A limitation of this article it is the retrospective nature of the paper. Even though the data from NTDB is a robust source for firearm injuries, it does not include data from acute care facilities that are not certified trauma centers, patients discharged from emergency departments, and does not speak to the circumstances preceding the injuries including homicide-suicide circumstances^6,7,37^. Our study also takes illicit and/or prescribed psychoactive substances into account, but did not evaluate the effect of alcohol. There is a breadth of literature discussing the relationship between ethanol use and both suicidality and homicides and may have confounded our data. While alcohol consumption is associated with increased suicide risk^46,47^, associations with alcohol and homicide relates to environment, situation, and variable consumption by assailant vs victim^47^, so we cannot infer that the patient who was shot as a bystander, assaulted, or shot in the course of other criminal activity was due to their own consumption. Although we are unable to perform a cost analysis, Cook et al. estimated the overall annual cost of firearm injuries to be $2.3 billion USD^60^. A more recent estimate of the societal cost of firearm injury ranks eighth on the US government expenditure list in 2010, with a cost of $174.1 billion USD^61^.

## Conclusions

This retrospective study found, firearm suicide injuries group had a higher in-hospital mortality and higher injury severity when compared to the firearm homicide group. Suicide attempts reaching the hospital alive tended to be older white males, have more frequent severe head and neck injuries, and more likely to have antidepressants detectable on routine drug screens. Homicide patients tended to be younger African-American males, have more extremity injuries, and found to have illicit/recreational substances detectable on drug screens. The majority of suicides and homicides were committed with handguns. Several risk factors for gun violence may be mitigated as clinicians during the index admission to assist in recovery.

## Data Availability

All data was obtained via the American College of Surgeons (ACS) National Trauma Database (NTDB). The datasets used and/or analyzed during the current study are available from the corresponding author on reasonable request. Data was obtained via the ACS NTDB website (https://www.facs.org/quality-programs/trauma/tqp/center-programs/ntdb).
